# A Structure-Based Deep Learning Framework for Correcting Marine Natural Products’ Misannotations Attributed to Host–Microbe Symbiosis

**DOI:** 10.3390/md24010020

**Published:** 2026-01-01

**Authors:** Xiaohe Tian, Chuanyu Lyu, Yiran Zhou, Liangren Zhang, Aili Fan, Zhenming Liu

**Affiliations:** State Key Laboratory of Natural and Biomimetic Drugs, School of Pharmaceutical Sciences, Peking University, Beijing 100191, China; xhtian@stu.pku.edu.cn (X.T.); cy.lyu@pku.edu.cn (C.L.); yrzhou@bjmu.edu.cn (Y.Z.); liangren@bjmu.edu.cn (L.Z.); fanaili@bjmu.edu.cn (A.F.)

**Keywords:** marine natural products, origin classification, cheminformatics, host-microbe symbiosis, AI-driven drug discovery

## Abstract

Marine natural products (MNPs) are a diverse group of bioactive compounds with varied chemical structures, but their biological origins are often misannotated due to complex host–microbe symbiosis. Propagated through public databases, such errors hinder biosynthetic studies and AI-driven drug discovery. Here, we develop a structure-based workflow of origin classification and misannotation correction for marine datasets. Using CMNPD and NPAtlas compounds, we integrate a two-step cleaning strategy that detects label inconsistencies and filters structural outliers with a microbial-pretrained graph neural network. The optimized model achieves a balanced accuracy of 85.56% and identifies 3996 compounds whose predicted microbial origins contradict their Animalia labels. These putative symbiotic metabolites cluster within known high-risk taxa, and interpretability analysis reveal biologically coherent structural patterns. This framework provides a scalable quality-control approach for natural product databases and supports more accurate biosynthetic gene cluster (BGC) tracing, host selection, and AI-driven marine natural product discovery.

## 1. Introduction

Natural products are secondary metabolites synthesized by organisms in nature. They possess diverse pharmacological activities and biological functions, conferring ecological advantages on survival or defense during evolution [1]. Covering approximately 70% of the Earth’s surface, the marine environment represents an immense reservoir of biological and chemical diversity. Compared with terrestrial habitats, the ocean is characterized by unique physicochemical conditions—including high pressure, high salinity, low temperature, large pH fluctuations, complex nutrient gradients, and limited light penetration—that drive the evolution of distinctive biosynthetic mechanisms [2]. As a result, marine natural products often display greater structural novelty and biological potency, representing an important source for drug discovery.

With advances in marine biology and microbiology, it has become increasingly evident that many natural products once attributed to marine invertebrates are, in fact, synthesized by their associated symbiotic microorganisms [3]. This phenomenon has redirected research efforts from macroorganisms toward marine microbes. However, the study of symbiotic microorganisms remains challenging. Most marine invertebrates are collected from natural environments, and their symbionts often fail to grow once removed from the host. Moreover, a substantial portion of marine microorganisms remain uncultivable under standard laboratory conditions, with cultivable taxa estimated to represent only a small fraction of the total microbial community [4]. Consequently, despite serving as a crucial source of marine natural products, symbiotic microorganisms are difficult to access experimentally, leading to a persistent high input but low output dilemma in marine natural product discovery. In this context, the ability to rapidly identify species with genuine biosynthetic potential has become essential for improving lead compound discovery efficiency and reducing wasted research efforts.

Early limitations in microbial cultivation techniques, combined with the complex symbiotic relationships between marine invertebrates and their associated microorganisms, have led to substantial uncertainty and potential misannotations in the reported biological origins of many marine natural products in both the literature and public databases. Major marine natural product databases (e.g., CMNPD [5]) compile compound entries and source information primarily from review articles and research reports. Thus, inaccuracies in the original literature often propagate into databases, leading to incorrect annotations for natural products. For instance, several compounds isolated from the Caribbean sponge *Smenospongia conulosa* were later found to resemble canonical cyanobacterial metabolites; yet, due to the lack of conclusive experimental evidence, they continued to be labeled as sponge-derived in reviews and databases [6].

As AI becomes increasingly integrated into natural product discovery, the consequences of misannotations are further amplified. Deep learning models are highly sensitive to training data quality. Systematic biases in databases inevitably lead to learned but incorrect structure–origin associations, resulting in wrong predictions. In the face of widespread misannotations caused by host–microbe symbiosis, developing tools that can systematically detect and correct erroneous labels is not only a matter of database curation, but also a critical prerequisite for improving the success rate of marine drug discovery, reducing unnecessary resource consumption, and enabling synthetic biology to transition from empirical trial-and-error to rational design.

The biosynthesis of natural products is typically orchestrated by modular enzymatic reactions. This modularity implies a latent relationship between the chemical structures of metabolites and their biological origins. In recent years, several studies have explored the feasibility of predicting natural product origins directly from molecular structures. For example, a study using the COCONUT database [7] employed MAP4 fingerprints [8] and a support vector machine (SVM) [9] classifier to accurately distinguish plant-, fungi-, and bacteria-derived natural products [10]. Similarly, Xu et al. developed a composite machine learning framework combining graph convolutional neural networks (GCNs) with gradient-boosted decision trees (XGBoost) [11], enabling the successful classification of five major biological origins: Plantae, Fungi, Chromista, Bacteria, and Animalia [12]. However, these models perform considerably worse on marine natural products, largely because marine organisms display far greater phylogenetic breadth and structural diversity than the terrestrial taxa used for training.

Based on the current situation, our study develops a marine-specific biological origin classifier trained on a systematically curated dataset of marine natural products. Unlike previous terrestrial-focused models, our framework is designed explicitly to address the profound label noise caused by host–microbe symbiosis in marine organisms. To this end, we integrate a microbial-pretrained graph neural network with a two-stage data-cleaning workflow. This marine-tailored strategy enables more reliable identification of potential misannotations directly from the molecular structure, thereby providing dependable guidance for compound isolation, database annotation, and biosynthetic gene cluster (BGC) source tracing. Ultimately, this work seeks to enhance database accuracy, reduce misleading experimental efforts, improve the overall efficiency of marine natural product exploration, and provide a robust data foundation for synthetic biology and AI-driven drug discovery.

## 2. Results and Discussion

### 2.1. Structural Characteristics of Marine Natural Products Support the Feasibility of Origin Classification

**Figure 1:**
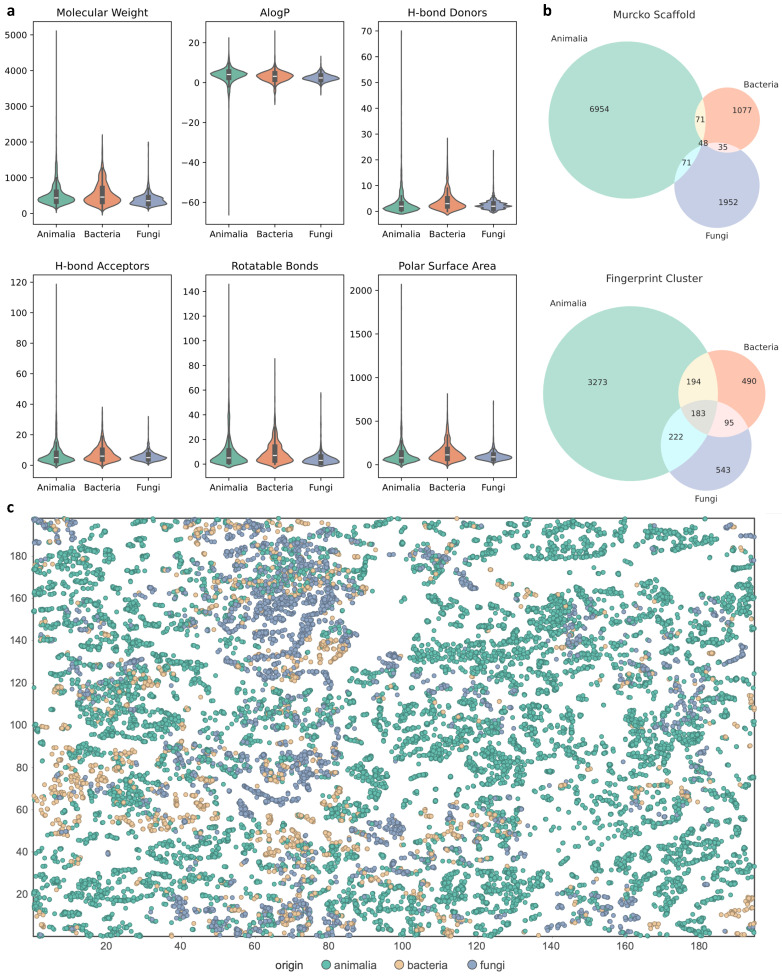
Cheminformatic analysis of the three major biological origins in CMNPD. (**a**) Distributions of physicochemical properties. (**b**) Overlap between scaffold- and fingerprint-based structural clusters. (**c**) SOM projection of chemical space.

A comprehensive cheminformatic analysis was first performed, as shown in Figure 1, on the three major source categories contained in the CMNPD database as these three categories account for 86% of the total.

The physicochemical property analysis revealed distinct trends across origins. Fungi-derived compounds showed moderate molecular weights (mostly less than 500 Da), balanced AlogP values (mostly less than 5), and low hydrogen bond donor (HBD) and acceptor (HBA) counts, making them the closest to Lipinski’s Rule of Five [13], which reflects membrane permeability, molecular flexibility, polarity, and other factors relevant to the drug-likeness of natural products. Bacterial metabolites tended to exhibit greater structural complexity, with higher numbers of HBD, HBA, and larger polar surface area (PSA), suggesting strong polarity and potential structural novelty. Animal-derived compounds showed the widest overall distribution, including numerous extreme values such as large macrocycles and polycyclic frameworks.

Scaffold- and fragment-level clustering further highlighted origin-dependent structural boundaries. Murcko scaffold [14] analysis showed minimal overlap between the three origins at the core-scaffold level, indicating strong structural separation. In contrast, Morgan-fingerprint [15] clustering demonstrated substantial overlap at the fragment level, suggesting that compounds from different origins may share functional substructures even when their core scaffolds differ.

To further examine the high-dimensional distribution of structural space, Self-Organizing Map (SOM) [16] visualization revealed additional inter-kingdom relationships. Bacterial and fungal natural products frequently occupied adjacent or overlapping regions, forming several dense structural clusters. Animal-derived compounds also overlapped broadly with microbial-enriched regions, consistent with the possibility of symbiosis-derived metabolites. Importantly, specific SOM regions showed clear structure–label mismatches, representing potential misannotation hotspots.

Collectively, these results support the feasibility of using structural information to distinguish biological origins and underscore the necessity of employing structure-based classification models for dataset cleaning and misannotation identification.

### 2.2. The Specialized Model Exhibits Superior Performance in the Marine Scenario

**Figure 2:**
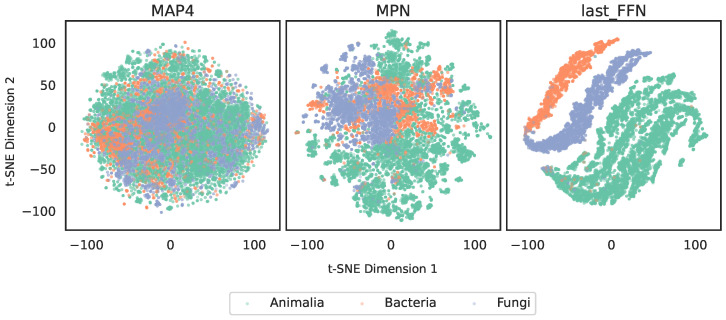
t-SNE visualization of molecular distributions based on three types of fingerprints.

Figure 2 shows the t-SNE [17] visualizations of molecular distributions using the three types of fingerprints.

The MAP4 fingerprint, as a traditional molecular descriptor, captures part of the inter-class distribution differences but displays substantial mixed regions, indicating limited discriminative power. The MPN fingerprint demonstrates clearer class-level aggregation: animal-derived compounds form a relatively distinct cluster, whereas bacteria and fungi still exhibit partial overlap. This suggests that the directed message passing neural network in the graph model captures structural patterns more effectively than handcrafted descriptors. The last_FFN fingerprint yields the most compact cluster separation, reflecting strong discriminative capability. This improvement likely benefits from supervised training signals introduced in the feed-forward neural network (FFNN) layers, enabling the learned embeddings to align more closely with classification-relevant structural features.

**Figure 3:**
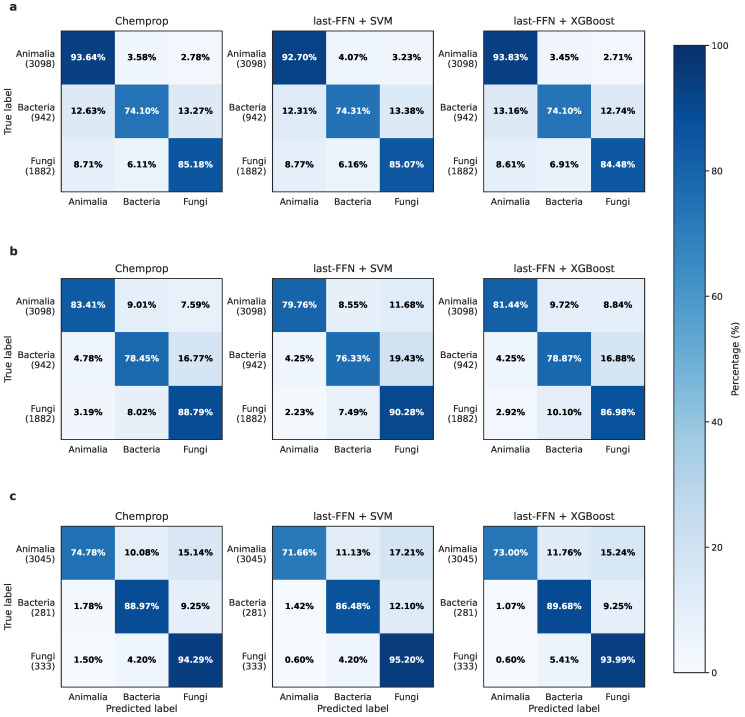
Confusion matrices of the nine models under three strategies. (**a**) Raw data; (**b**) data after two-step cleaning; (**c**) final results after microbial pretraining.

Across the nine models constructed under different data-processing strategies, both the data-cleaning pipeline and microbial pretraining markedly improved origin-classification performance. As shown in Figure 3 and Table 1, we calculated the classification accuracy for each model and the balanced accuracy (*N*: total number of classes; $TP_{i}$: true positives for class *i*; and $FN_{i}$: false negatives for class *i*):

(1) $$\mathrm{Balanced}\;\mathrm{Accuracy}=\frac{1}{N}\sum_{i=1}^{N}\frac{TP_{i}}{TP_{i}+FN_{i}}$$

Using the raw dataset, the Chemprop [18] model achieved a balanced accuracy of 84.31% on the 5922-compound test set. The Animalia class reached the highest accuracy (93.64%), followed by Fungi (85.18%). Bacteria, however, showed the lowest accuracy (74.10%), with many samples misclassified as Animalia or Fungi, indicating blurred structural boundaries. Training SVM and XGBoost classifiers on last_FFN embeddings produced performance comparable to the GCN model, with no substantial improvement observed.

On the cleaned dataset, all models exhibited clear performance gains on microorganisms. Fungal samples were identified with the highest accuracy, while boundaries between the two microbial groups remained partially ambiguous, suggesting intrinsic structural overlap. The apparent decrease in Animalia accuracy is primarily attributable to the substantial misannotations within the test set itself. Because the test set still contains a large number of Animalia-labeled compounds whose structures resemble microbial metabolites, a cleaner and more stringent model will correctly classify these samples as microbial, which is counted as an error under the noisy ground-truth labels. Thus, the reduced accuracy does not reflect impaired model performance, but instead highlights the extensive label noise in CMNPD2.0 and further validates the necessity of a dedicated cleaning pipeline.

The combination of microbial pretraining and cleaned data further enhanced the model’s ability to capture microbial structural patterns. After incorporating microbial pretraining, the last_FFN + XGBoost model achieved pronounced improvements: Bacteria accuracy increased from 78.87% to 89.68%; Fungi accuracy increased to 93.99%, yielding an overall balanced accuracy of 85.56%. These gains confirm the effectiveness of microbial-informed representation learning. Although the Animalia accuracy slightly decreased, this is consistent with the substantial removal of potential misannotations during the cleaning process and reflects a more biologically realistic structural interpretation.

From a modular perspective, the observed performance improvements can be attributed to complementary contributions from different components of the framework. The Chemprop backbone provides a robust molecular encoder that captures general chemical features, while the task-specific classification heads further adapt these representations to origin classification. The consistent performance across different classifiers on the same embeddings indicates that the learned representation, rather than the choice of downstream classifier, is the primary determinant of model performance.

**Figure 4:**
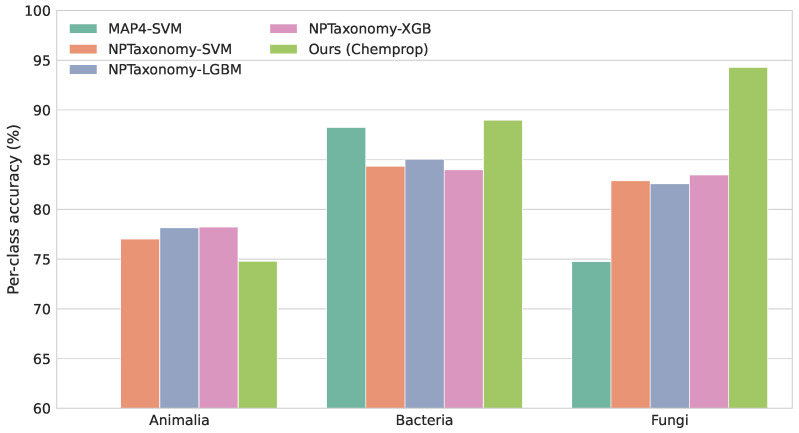
Per-class accuracy comparison between the proposed framework and two previously published natural product origin classification models on overlapping categories.

To further evaluate the performance of our framework, we compared it with two previously published representative models for natural product origin classification [10,12]. We note that these reference models were primarily trained on terrestrial natural product datasets and were designed for different taxonomic classification schemes, which are not fully aligned with the marine origin classification task addressed in this study. Consequently, a direct comparison across all categories is not feasible. To enable a fair and informative evaluation, we therefore restricted the comparison to the overlapping origin categories shared by these models and our framework, and assessed performance using per-class accuracy. As shown in Figure 4, our model achieves competitive accuracy across the shared categories. In particular, for microbial-associated classes, our framework demonstrates comparable or improved classification performance relative to the reference models, highlighting its ability to capture microbial structural features even when compared against models trained on larger, predominantly terrestrial datasets. These results indicate that the proposed framework generalizes well across shared categories while providing improved specificity for marine-origin compounds.

### 2.3. Two-Step Cleaning Strategy Substantially Improves Data Quality

#### 2.3.1. Cleaning Outcomes

**Figure 5:**
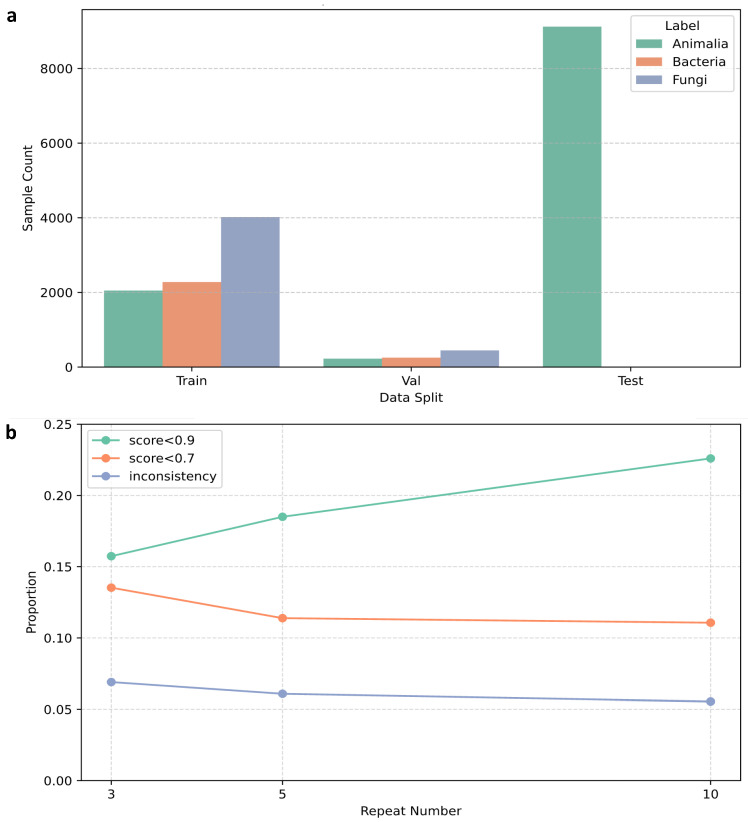
Cross-prediction cleaning workflow. (**a**) Category composition of the training set in each iteration. (**b**) Confidence scores and inconsistency proportions for Animalia samples under different repetition numbers.

Category distribution and prediction confidence across repeated cross-prediction cycles are shown in Figure 5. Across ten rounds of repeated predictions, a total of 632 Animalia-labeled compounds (approximately 5.5%) were identified as exhibiting clear label–structure inconsistencies, with most being recurrently predicted as microbial in origin.

Using the last_FFN embeddings, a KDTree [19] was constructed and the resulting structure space was visualized with t-SNE. The visualization revealed well-defined clusters for bacterial and fungal metabolites. Notably, a small number of bacterial or fungal samples were positioned inside the Animalia cluster, indicating localized inconsistencies in the structural space. After applying a neighborhood-consistency filter, 617 structurally ambiguous samples were further removed. After the two-step cleaning, the model was retrained on the cleaned dataset.

#### 2.3.2. Distribution of Putative Symbiotic Marine Metabolites

Based on the final pretrained last_FFN + XGBoost model, 3996 compounds annotated as Animalia in CMNPD were identified as putative symbiotic metabolites (in Supplementary Materials), meaning that their predicted origin conflicted with the annotated Animalia label.

**Figure 6:**
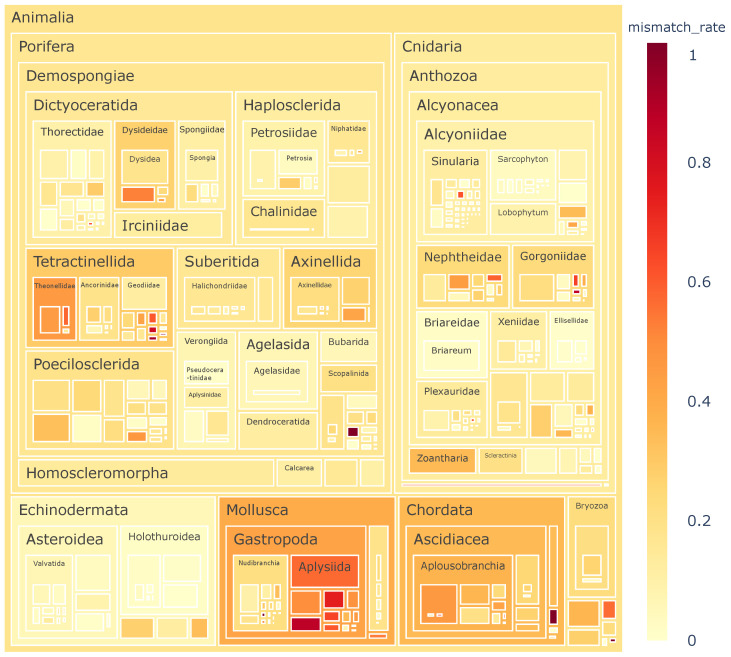
Species distribution of putative symbiotic marine metabolites. To further facilitate detailed inspection, an interactive html version of the figure is included in the Supplementary Materials.

The tree map of Figure 6 revealed strong aggregation of these putative symbiotic metabolites within specific animal phyla, suggesting that certain taxonomic groups may have a higher dependency on microbial symbionts, or that systematic annotation biases exist in these lineages within the database. For example, members of Mollusca, particularly Aplysiida, form prominent clusters of suspected misannotations. This observation is consistent with the literature: Dolastatin 10, first discovered in 1988 and initially attributed to the animal host Aplysiida [20], was later isolated from cyanobacteria [21], demonstrating that Aplysiida represents a high-risk group for mislabeling due to their symbiotic relationships.

### 2.4. Case Studies

**Figure 7:**
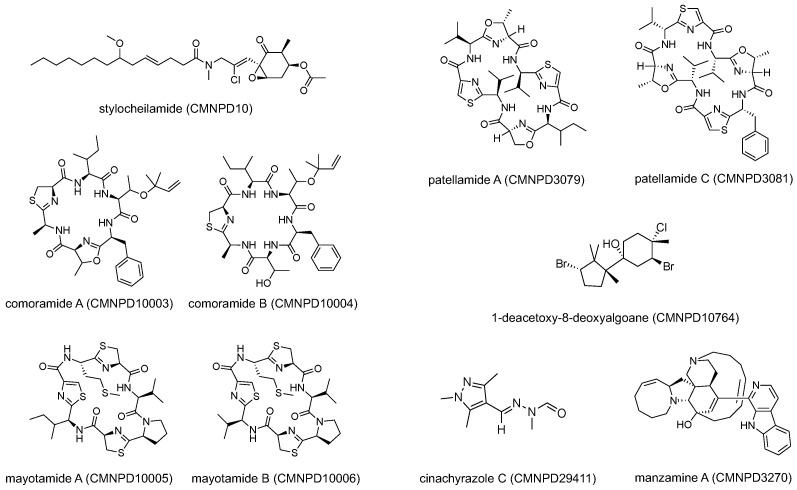
Case studies of putative misannotated structures.

To verify the biological credibility of the model’s predictions, we performed literature tracing and structural verification for high-confidence misannotated candidates in Figure 7.

Among them, stylocheilamide (CMNPD10) possesses an unusual scaffold with multiple substituents. The original report noted that such features raise the question of the biogenesis and the biological origin [22], and ultimately attributed the compound to symbiotic organisms such as red algae or cyanobacteria ingested by the Aplysiida. Similarly, 1-deacetoxy-8-deoxyalgoane (CMNPD10764) represents another example of origin ambiguity in Aplysiida-derived metabolites. This halogenated sesquiterpene was isolated from the digestive glands of *Aplysia dactylomela*. The authors explicitly noted that the suspected red algal sources of these compounds remain unidentified, suggesting a dietary or symbiotic origin rather than de novo biosynthesis by the animal host [23]. Together with stylocheilamide, these cases highlight Aplysiida as a high-risk group for origin misannotation driven by symbiotic or dietary metabolite sequestration.

Comoramide A (CMNPD10003), comoramide B (CMNPD10004), mayotamide A (CMNPD10005), and mayotamide B (CMNPD10006) are four peptide natural products labeled as animal-derived compounds in CMNPD. However, their chemical structures deviate substantially from classical animal-derived peptides. The original literature explicitly suggests that these compounds are likely synthesized by symbiotic prokaryotic cyanobacteria, such as the genus *Prochloron*, consistent with related peptide families [24]. These observations strongly indicate that their true origin is a microorganism rather than an animal.

Patellamide A (CMNPD3079) and patellamide C (CMNPD3081) represent another well-established example of symbiosis-associated marine natural products. These cyclic peptides were originally isolated from the ascidian *Lissoclinum patella* and consequently annotated as animal-derived compounds in early literature and public databases [25]. However, subsequent biosynthetic investigations demonstrated that patellamides are synthesized by the obligate cyanobacterial symbiont *Prochloron didemni* rather than by the ascidian host itself. Genomic analysis and heterologous expression experiments confirmed that the complete patellamide biosynthetic gene cluster is encoded in the symbiont genome and operates via a microcin-like pathway [26].

Cinachyrazole C (CMNPD29411) represents another example. Pyrazole derivatives of cinachyrazoles A–C were originally isolated from the marine sponge *Cinachyrella* sp. [27]. However, the authors explicitly suggested that cinachyrazole C may be derived from a fungal symbiont-related metabolite, namely N-formyl-N-methylhydrazine, which was previously isolated from the ascomycete *Gyromitra esculenta* [28]. This biosynthetic hypothesis implies that cinachyrazole C is more likely of microbial rather than animal origin.

The above cases were all successfully predicted by our model as microbial-associated rather than animal-derived, despite their original database annotations. These results demonstrate the potential of our framework to identify symbiosis-related natural products and to flag compounds with ambiguous or potentially misannotated origins based solely on structural information. However, we also note that not all classical symbiosis-associated compounds were correctly reassigned.

Manzamine A (CMNPD3270) is a well-known antimalarial alkaloid originally isolated from the marine sponge [29], but later studies demonstrated that it can be produced by its symbiotic bacterium *Micromonospora* sp. [30]. However, it was classified by our model as Animalia-derived. A post hoc analysis showed that multiple compounds with high structural similarity to manzamine A (Tanimoto similarity > 0.7; CMNPD5448, CMNPD6811, CMNPD11949, CMNPD29421, CMNPD8205, CMNPD9689, CMNPD3270, CMNPD2722, CMNPD3271, and CMNPD13465) are all annotated as Animalia-derived in public databases. As a result, the local structural neighborhood of manzamine A in the learned embedding space is dominated by Animalia-labeled samples, leading to an Animalia prediction in this strictly structure-based, data-driven framework. This case illustrates how long-standing annotation practices in marine symbiotic systems can introduce systematic bias, underscoring the importance of cleaner and more accurately curated training datasets, which will be a key focus of our future work.

### 2.5. Interpretability Analysis

#### 2.5.1. Chemical Scaffolds

**Figure 8:**
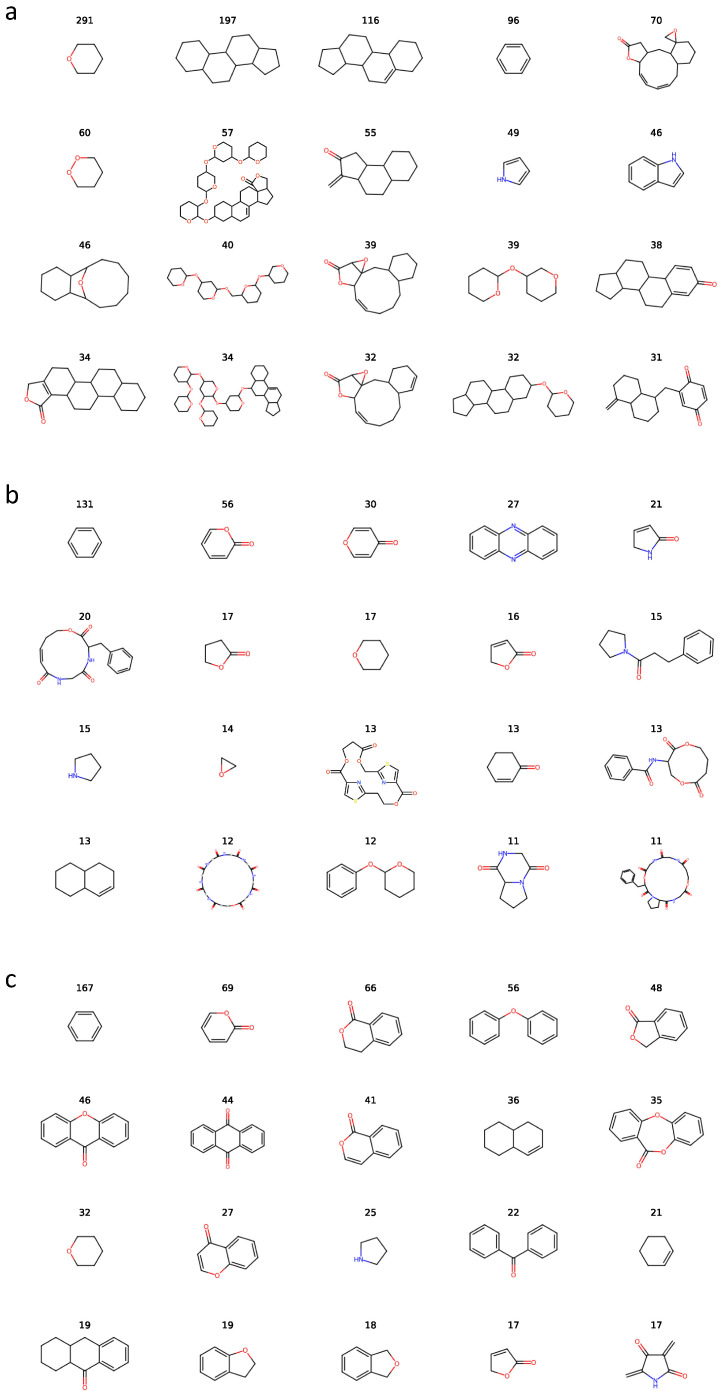
Counts of representative Murcko scaffolds of the three biological kingdoms: (**a**) Animalia, (**b**) Bacteria, and (**c**) Fungi. Atoms are colored according to different elements.

Murcko scaffold analysis in Figure 8 revealed clear structural differences among kingdoms. Animal-derived natural products are dominated by steroidal and other saturated five- and six-membered ring systems, whereas microbial metabolites display a broader diversity of aromatic and heterocyclic scaffolds. Within the microbial group, bacterial compounds are strongly enriched in nitrogen-containing heterocycles, while fungal metabolites more frequently feature tricyclic and polyketide-derived frameworks.

However, Murcko scaffolds also present methodological limitations. Because the scheme strictly preserves only the ring system and linker skeleton, even minor structural modifications produce distinct scaffolds, artificially reducing overlap and affecting interpretability. Furthermore, highly common motifs such as benzene rings are recorded as independent scaffolds despite offering limited value for origin differentiation. Thus, complementary analysis using more origin-relevant structural descriptors are necessary.

#### 2.5.2. Biosynthetic Pathways

**Figure 9:**
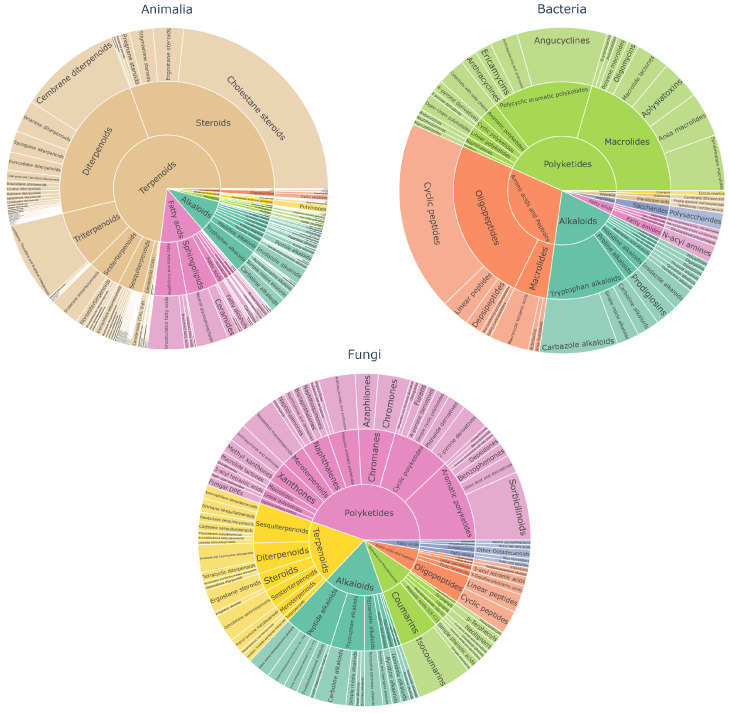
Predicted biosynthetic pathways for the three biological kingdoms. Interactive html versions of the pathway distributions for all three origin categories are available in the Supplementary Materials.

The biosynthetic pathway annotations in Figure 9 showed that Animal-derived compounds are dominated by terpenoids, particularly sesquiterpenes, diterpenes, triterpenes and steroids, which appear at substantially higher proportions than in microbial samples. Bacterial metabolites are enriched in polyketides and peptides (especially small oligopeptides). Fungal metabolites exhibit broader pathway diversity but are largely dominated by polyketides and terpenoids.

It is important to note that NPClassifier [31] is a neural-network-based structural classifier, inferring pathways from chemical features rather than experimental biosynthetic evidence. While uncertainties remain for highly novel structures, the large-scale trends remain informative. Moreover, the predicted biosynthetic classes are broadly consistent with the scaffold-level observations, further confirming the structural and biosynthetic divergence among the three kingdoms.

#### 2.5.3. Fragment Contribution Analysis Based on MCTS

**Figure 10:**
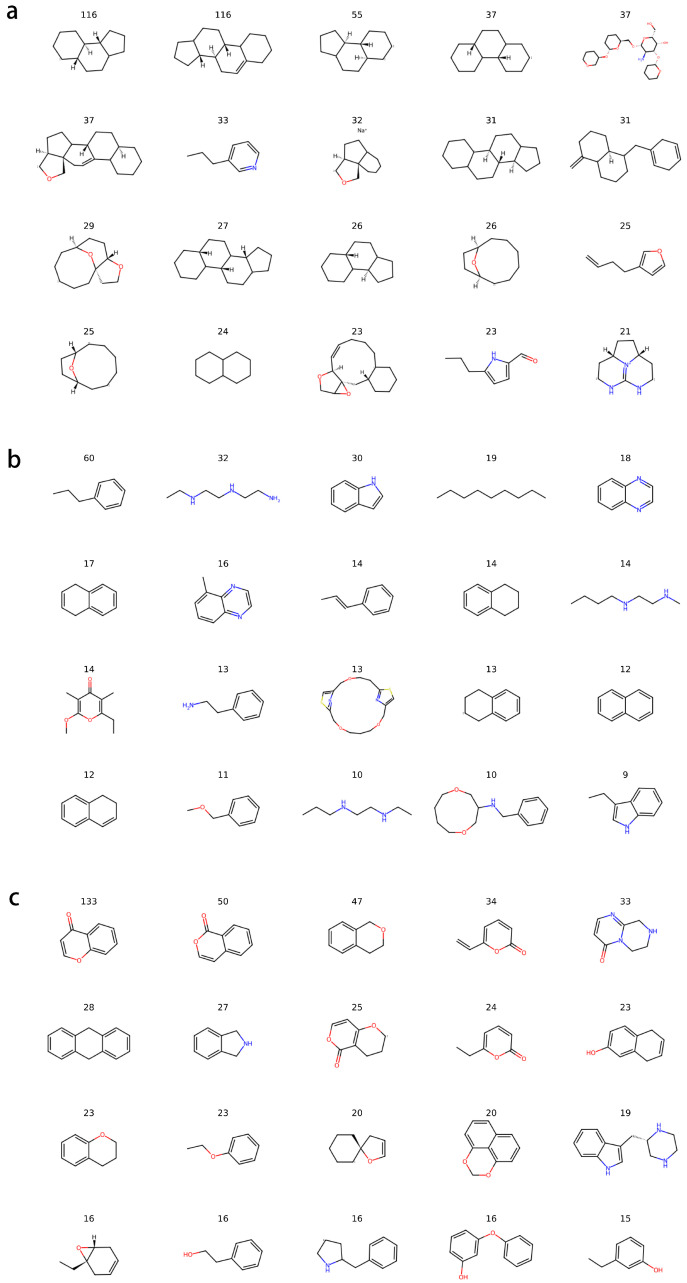
Counts of rationales revealed by MCTS of the three biological kingdoms: (**a**) Animalia, (**b**) Bacteria, and (**c**) Fungi. Atoms are colored according to different elements.

The rationales extracted using Monte Carlo Tree Search (MCTS) revealed clear structural signatures across biological origins in Figure 10. Animal-derived natural products were characterized by steroidal cores and fused-ring systems. Bacterial metabolites frequently contained nitrogen heterocycles and aromatic systems. Fungal metabolites exhibited abundant carbonyl groups, ester linkages, and other characteristic polyketide motifs.

Compared with murcko scaffolds, MCTS identifies minimal substructures that preserve classification confidence, focusing on fine-grained structural determinants most relevant to model decisions. This aligns closely with the mechanism of graph neural networks, which operate on local neighborhood information. Therefore, MCTS provides higher interpretability and flexibility, particularly within the structurally diverse natural product chemical space.

## 3. Materials and Methods

### 3.1. Data Collection and Preprocessing

Two major natural product databases were used in this study:

- CMNPD: Compounds annotated as originating from Animalia, Bacteria, and Fungi were extracted from CMNPD version 1.0 (www.cmnpd.org, accessed on 13 September 2025) and version 2.0 (temporarily unavailable on website but provided in Supplementary Materials). From CMNPD 1.0, a total of 18,402 molecules published after the year 2000 were selected as the training set, comprising 11,402 Animalia, 2532 Bacteria, and 4468 Fungi entries. The test set consisted of 5922 non-overlapping compounds retrieved from CMNPD 2.0, including 3098 Animalia, 1882 Bacteria, and 942 Fungi samples.

- NPAtlas [32]: NPAtlas was used as a high-confidence reference for pretraining microbial structural features. It contains 22,995 fungal natural products and 13,459 bacterial natural products with biological origin annotations and is publicly available at www.npatlas.org (accessed on 6 July 2025).

All biological origin annotations were mapped to the Kingdom level using a three-class scheme: 0 = Animalia, 1 = Bacteria, and 2 = Fungi. Molecular structures were represented using SMILES strings for subsequent feature extraction and model training.

### 3.2. Cheminformatic Analysis

#### 3.2.1. Physicochemical Property Analysis

The physicochemical properties of CMNPD compounds were evaluated using RDKit [33]. Six drug-likeness-associated descriptors were selected, including molecular weight (MW), AlogP, H-bond donors, H-bond acceptors, rotatable bonds, and polar surface area.

#### 3.2.2. Scaffold Extraction and Fragment-Based Clustering

To investigate structural similarities and differences across biological origins, scaffold extraction and fragment-based clustering were performed using RDKit:

- Murcko scaffold extraction: side chains were removed and only the core ring systems and linkers were retained to characterize scaffold-level diversity.
- Morgan fingerprint (Extended-Connectivity Fingerprints, ECFP) encoding: fingerprints of 1024 bits were generated using a radius of 6 to capture local atomic environments.
- Dimensionality reduction: principal component analysis (PCA) [34] was applied in scikit-learn [35] to compress Morgan fingerprints to 85 dimensions to reduce computational cost.
- Clustering: all compounds were clustered into 5000 structural groups using the K-means algorithm [36] implemented in scikit-learn.
- Structural overlap assessment: Venn diagrams were generated to evaluate cross-kingdom structural overlap under both Murcko scaffolds and Morgan fingerprint clusters.

#### 3.2.3. Chemical Space Visualization and Cross-Kingdom Mixing Analysis

To visualize the distribution of marine natural products in high-dimensional structural space, Self-Organizing Map (SOM) projections were generated using the SOM module in DataWarrior [37], based on SkelSpheres descriptors. The SOM grid size was set to 200 × 200, as this resolution provides the best dispersion of structural clusters while substantially reducing computation time and cost compared with larger grid sizes. Each compound was colored according to its original biological origin annotation.

### 3.3. Molecular Representations and Feature Extraction

**Figure 11:**
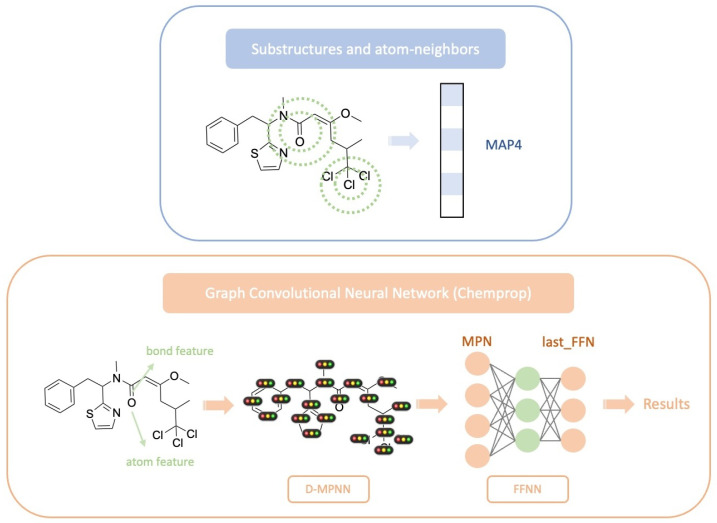
Three molecular fingerprint representations.

Three molecular fingerprints and representations in Figure 11 were evaluated for subsequent classification modeling and structural clustering:

- MAP4 fingerprint: A 1024-dimensional descriptor integrating atom–connection patterns and topological path information, suitable for modeling the structural diversity of natural products.
- MPN representation: Intermediate embeddings extracted from the directed message passing neural network (D-MPNN) encoder implemented in the Chemprop framework (default dimensionality: 1100).
- last_FFN representation: The final embedding obtained from the last fully connected layer of the feed-forward neural network (FFNN) following the D-MPNN encoder (default dimensionality: 1100).

Comparison using t-SNE visualization demonstrated that the last_FFN representation provides the best separation between microbial and animal-derived samples. Therefore, it was adopted as the primary molecular representation for all subsequent analysis.

### 3.4. Construction and Training of the Classification Model

The GCN model was implemented using Chemprop, in which molecular structures are encoded with a directed message passing neural network (D-MPNN) followed by a feed-forward neural network (FFNN) for classification.

The overall modeling framework consists of two stages:

- Pretraining Stage: We first pretrained the D-MPNN encoder on a combined dataset consisting of CMNPD animal-derived compounds and an equal-sized subset of microbially derived compounds from NPAtlas. This procedure enabled the encoder to learn generalizable structure embeddings across the three kingdoms and to obtain robust microbial structural representations.
- Fine-tuning Stage: During fine-tuning on the CMNPD training set, the pretrained D-MPNN encoder was frozen, and only the FFNN classifier was updated. This strategy prevents the model from overfitting to noisy or potentially misannotated samples in the small CMNPD dataset.

To further improve model stability and interpretability, the last_FFN embeddings generated in each training round were extracted and used to train two additional classical machine-learning models independently: SVM and XGBoost. Classification performance was evaluated using confusion matrix, which quantifies per-class accuracy and misclassification patterns across Animalia, Bacteria, and Fungi.

### 3.5. Misannotation Identification and Data-Cleaning Strategy

The annotation of marine natural products has long been affected by two major errors: subjective inference, in which researchers assigned origins based mainly on host; and intrinsic ambiguity in chemical space, where metabolites derived from symbiotic systems may exhibit structural features of both microbial and animal sources. To address errors attributed to these two levels, we designed a two-step cleaning workflow.

#### 3.5.1. Misannotation Screening via Cross-Prediction

Since there is no universally accepted gold-standard annotation for natural product origins, we adopted a label-correction strategy inspired by machine learning practices using repeated cross-prediction cycles [38].

In each training iteration, a randomly selected subset of animal-derived compounds was combined with all microbial samples (bacterial and fungal) to form the training set. A classifier was trained on this subset, and the remaining animal samples were treated as an out-of-fold (OOF) validation set for prediction. This procedure was repeated multiple times. For each animal compound, its OOF predictions were aggregated and averaged across all cycles. If the averaged prediction conflicted with its original database label, the compound was flagged as a putative misannotation. The rationale is that animal-derived annotations in historical literature often rely on experience-based judgments and are therefore more prone to error, whereas microbial origins are generally supported by direct isolation and are more reliable. Repeated model training with varying subsets allows potential misannotations to be detected automatically, without manual supervision or a gold-standard reference.

**Figure 12:**
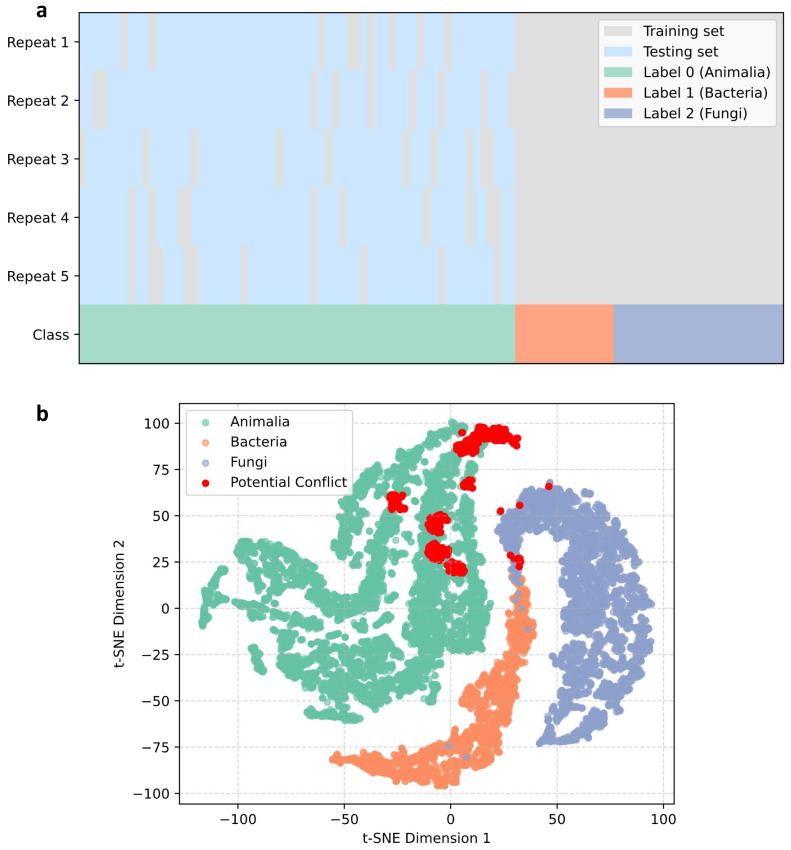
Illustration of the two-step procedure: (**a**) misannotation screening via cross-prediction; (**b**) removal of structurally inconsistent samples based on neighborhood analysis.

As illustrated in Figure 12a, the gray region represents the training subset for each iteration, and the blue region represents the samples held out for prediction. This procedure was applied to the initial dataset containing 18,402 compounds. Repeated resampling ensured coverage across Animalia, Bacteria, and Fungi, partially mitigating class imbalance during training.

#### 3.5.2. Removal of Structurally Inconsistent Samples Based on Neighborhood Analysis

To further ensure the reliability of the training set, we retrained the GCN model using the data remaining after Step 1 and extracted the last_FFN embeddings as structural fingerprints for each compound. These embeddings were visualized using t-SNE, and a KDTree index was constructed from the full embedding space to enable efficient local-neighborhood queries. Using a radius threshold of 5.0, chosen based on the observed distance distribution of samples in the embedding space, we identified and removed Animalia-annotated samples that lay within close structural proximity to certain bacterial or fungal samples. These Animalia points were considered likely misannotated and were removed to prevent local microbial clusters from being distorted during model training, as shown in Figure 12b.

### 3.6. Visualization and Model Interpretability Analysis

#### 3.6.1. Visualization of Scaffold-Level Features

To examine the core structural features associated with different biological origins, Murcko scaffolds were extracted for all compounds in the final cleaned training set. For each of the three classes, the top 20 most frequent Murcko scaffolds were computed, and their counts were visualized to compare scaffold usage across kingdoms.

#### 3.6.2. Biosynthetic-Pathway-Based Structural Classification

To biologically contextualize structural differences between origins, the NPClassifier tool was used to annotate the putative biosynthetic pathways of all compounds in the training set. NPClassifier is a deep learning-based framework that predicts biosynthetic classes such as terpenes, polyketides, peptides, and others. The predicted pathway categories were summarized by biological origin, and the pathway compositions were visualized to highlight inter-kingdom differences in biosynthetic tendencies.

#### 3.6.3. Model Interpretability via Monte Carlo Tree Search (MCTS)

To identify structural features most critical to the model’s classification decisions, interpretability analysis was performed using the interpret module of Chemprop, integrated with a Monte Carlo Tree Search (MCTS) procedure. MCTS is a heuristic search algorithm that iteratively selects, expands, simulates, and backtracks through nodes in a complex structural space. In this study, MCTS was applied to each compound in the training set by progressively modifying the parent molecule to generate a set of substructures (rationales), achieved through selectively removing or retaining specific fragments.

Each substructure was evaluated by the trained classifier to determine whether it maintained a prediction score above a predefined threshold for the corresponding biological origin. Through repeated iterations, the MCTS search converged toward a set of minimal predictive substructures, representing the model-identified key structural determinants. Finally, rationales from Animalia-, Bacteria-, and Fungi-derived samples were aggregated, clustered, and visualized to reveal common structural motifs and origin-specific predictive features.

## 4. Conclusions

In this study, we present a systematic framework specifically designed for origin classification and misannotation correction in marine symbiotic natural product systems. By integrating deep learning modeling, structural-space visualization, confidence-based prediction assessment, and multi-level structural interpretability, the workflow enables the reliable detection and correction of potential labeling errors in marine natural product databases.

Furthermore, the distribution patterns of the putative symbiotic metabolites identified in this work are highly consistent with well-established ecological knowledge of marine symbiosis, underscoring the biological relevance of the model’s predictions. High-quality and trustworthy origin annotations are critical not only for understanding the chemical diversity of marine natural products, but also for downstream applications in synthetic biology and metabolic engineering. From a broader perspective, the structure-driven correction framework established in this work has the potential to become a key component of next-generation intelligent natural product discovery platforms. As artificial intelligence is increasingly applied to natural product, data quality is rapidly emerging as the primary determinant of discovery efficiency. The workflow proposed here not only offers a rigorous methodological basis for database quality control, but also lays a foundation for large-scale data mining, high-precision annotation, and AI-enabled drug discovery in marine natural product research.

However, the proposed framework is computational and structure-based in nature, and direct experimental validation of individual origin reassignment cases is beyond the scope of this study. In addition, the model depends on the quality and representativeness of available training data, and the current taxonomic coverage may not fully capture the diversity and fine-grained distinctions across all marine lineages. Moreover, the present implementation focuses on a specific deep learning architecture, and the exploration of additional model designs may further improve performance and generalizability. Future work will therefore aim to integrate targeted experimental validation, expand taxonomic coverage, and evaluate alternative model architectures to enhance the robustness and applicability of the framework.

## Figures and Tables

**Table 1:** Performance comparison across nine models under different data-processing strategies.

Data	Model	Balanced Acc	Ani Acc	Ani to Bac	Ani to Fun	Bac Acc	Bac to Ani	Bac to Fun	Fun Acc	Fun to Ani	Fun to Bac
Raw	GCN (Chemprop)	84.31%	93.64%	3.58%	2.78%	74.10%	12.63%	13.27%	85.18%	8.71%	6.11%
	last_FFN + SVM	84.03%	92.70%	4.07%	3.23%	74.31%	12.31%	13.38%	85.07%	8.77%	6.16%
	last_FFN + XGBoost	84.14%	93.83%	3.45%	2.71%	74.10%	13.16%	12.74%	84.48%	8.61%	6.91%
Cleaned	GCN (Chemprop)	83.55%	83.41%	9.01%	7.59%	78.45%	4.78%	16.77%	88.79%	3.19%	8.02%
	last_FFN + SVM	82.12%	79.76%	8.55%	11.68%	76.33%	4.25%	19.43%	90.28%	2.23%	7.49%
	last_FFN + XGBoost	82.43%	81.44%	9.72%	8.84%	78.87%	4.25%	16.88%	86.98%	2.92%	10.10%
Pretrained	GCN (Chemprop)	86.01%	74.78%	10.08%	15.14%	88.97%	1.78%	9.25%	94.29%	1.50%	4.20%
	last_FFN + SVM	84.45%	71.66%	11.13%	17.21%	86.48%	1.42%	12.10%	95.20%	0.60%	4.20%
	last_FFN + XGBoost	85.56%	73.00%	11.76%	15.24%	89.68%	1.07%	9.25%	93.99%	0.60%	5.41%

Abbreviations: Ani = Animalia; Bac = Bacteria; Fun = Fungi; Acc = Accuracy.

## Data Availability

The data and code of this study are available on https://github.com/xhhhher/MNP-Taxonomy-Classifier (accessed on 29 December 2025).

## Supplementary Materials

The following supporting information can be downloaded at https://www.mdpi.com/article/10.3390/md24010020/s1, Table S1: Test set from CMNPD2.0; Table S2: Putative symbiotic metabolites predicted by our model; Figure S1: Interactive treemap visualization showing the full taxonomic hierarchy of compounds annotated as Animalia in CMNPD, colored by mismatch rate; Figures S2–S4: Interactive sunburst visualizations of biosynthetic pathway annotations for compounds annotated as Animalia, Bacteria and Fungi.

## Abbreviations

The following abbreviations are used in this manuscript:

AI	Artificial Intelligence
BGC	Biosynthetic Gene Cluster
D-MPNN	Directed Message Passing Neural Network
ECFP	Extended-Connectivity Fingerprints
FFNN	Feed-Forward Neural Network
GCN	Graph Convolutional Neural Network
HBA	H-Bond Acceptor
HBD	H-Bond Donor
MCTS	Monte Carlo Tree Search
MNP	Marine Natural Products
MW	Molecular Weight
OOF	Out-of-Fold
PCA	Principal Component Analysis
PSA	Polar Surface Area
SOM	Self-Organizing Map
SVM	Support Vector Machine
XGBoost	Extreme Gradient Boosting
